# On the Versatile Role of Electrospun Polymer Nanofibers as Photocatalytic Hybrid Materials Applied to Contaminated Water Remediation: A Brief Review

**DOI:** 10.3390/nano12050756

**Published:** 2022-02-24

**Authors:** Alexander Cordoba, Cesar Saldias, Marcela Urzúa, Marco Montalti, Moreno Guernelli, Maria Letizia Focarete, Angel Leiva

**Affiliations:** 1 Department of Physical Chemistry, Faculty of Chemistry and Pharmacy, Pontifical Catholic University of Chile, Santiago 7820436, Chile; acordoba@uc.cl (A.C.); casaldia@uc.cl (C.S.); 2 Department of Chemistry “Giacomo Ciamician” and National Consortium of Materials Science and Technology (I.N.S.T.M., Bologna RU), Alma Mater Studiorum–Università di Bologna, 40126 Bologna, Italy; marco.montalti2@unibo.it (M.M.); moreno.guernelli2@unibo.it (M.G.); 3 Department of Chemistry, Faculty of Sciences, University of Chile, Santiago 7820436, Chile; maurzua@uchile.cl; 4 Health Sciences and Technologies-Interdepartmental Center for Industrial Research, Alma Mater Studiorum–Università di Bologna, 40126 Bologna, Italy

**Keywords:** polymer nanofibers, electrospinning, composite nanofibers, photocatalytic hybrid materials, water remediation

## Abstract

A wide variety of materials, strategies, and methods have been proposed to face the challenge of wastewater pollution. The most innovative and promising approaches include the hybrid materials made of polymeric nanofibers and photocatalytic nanoparticles. Electrospun nanofibers with unique properties, such as nanosized diameter, large specific surface area, and high aspect ratio, represent promising materials to support and stabilize photocatalytic nanosized semiconductors. Additionally, the role performed by polymer nanofibers can be extended even further since they can act as an active medium for the in situ synthesis of photocatalytic metal nanoparticles or contribute to pollutant adsorption, facilitating their approach to the photocatalytic sites and their subsequent photodegradation. In this paper, we review the state of the art of electrospun polymer/semiconductor hybrid nanofibers possessing photocatalytic activity and used for the remediation of polluted water by light-driven processes (i.e., based on photocatalytic activity). The crucial role of polymer nanofibers and their versatility in these types of procedures are emphasized.

## 1. Introduction

Nowadays, one of the most critical problems facing humanity is the increasing water contamination, mainly attributed to the negative impact of anthropogenic activities on varied aquatic environments. It is well-documented that skillful and environmentally friendly polluted water and wastewater management has a pivotal role in effectively mitigating adverse environmental impacts and the availability of, e.g., freshwater sources, especially in less developed countries. In recent years, an increasing number of pollutants, such as dyes, pharmaceuticals, pesticides, and other organic molecules, have been detected in water sources. The wastewater treatments available to date, such as coagulation, flocculation, or adsorption, merely concentrate or transfer the contaminants from one phase to another without their complete elimination [1].

In this context, advanced oxidation processes emerge as an innovative green chemical strategy to degrade the main organic pollutants present in wastewater. Interestingly, this type of process could involve photocatalytic reactions that generate highly reactive oxygen species (e.g., OH, O_2_, O_3_, and H_2_O_2_) [2]. The photogenerated reactive oxygen species can completely degrade most organic pollutants, bacteria, and viruses. The used photocatalysts are commonly based on nanosized semiconductor compounds (e.g., ZnO, CuO, TiO_2_, Fe_2_O_3_, CdS, PdS, AgBr, and ZnS) [3]. Using these nanomaterials makes it feasible to simultaneously take advantage of the semiconductor photo-properties and their high active surface area. These attributes strongly contribute to improve the efficiency of light-driven processes [4]. However, a decrease in the photocatalytic activity of nanoparticles (NPs) has been observed after a certain number of cycles of use, an effect which is essentially ascribed to their tendency to agglomerate, reducing the effective surface area spontaneously. Moreover, the efficient recovery of NPs from the reaction medium for subsequent reuse has also been a challenging task to date [4].

In order to solve these drawbacks, different types of structural supports for nanoparticles have been proposed. Glass mats, inorganic-carbon fibers, polymers, among others, are some materials typically used [2]. Electrospun polymer nanofibers (NFs) have emerged as a promising alternative to support and stabilize NPs in many chemical environments, preserving their highly valuable properties over time and after repeated use cycles [5]. Materials composed by NFs display unique functional properties, such as nanosized diameter, large specific surface area, high aspect ratio, high degree of porosity, and pore interconnectivity. These materials can be obtained as macroscopic mat-like structures, characterized by flexibility and elasticity, which can be advantageously used as a platform for numerous emerging environmental applications, e.g., the filtration of liquids, separation of fine particles for water treatment and technology environmental remediation processes, as well as, as photocatalyst support [6]. The high surface-to-volume ratio and continuous matrix structure, characterized by small pores, confer to these materials several advantages in the separation process of contaminants, especially when the surface/interface interactions play an essential role [6]. Additionally, another significant advantage is the enormous surface modification possibilities that polymer nanofibers offer [7,8]. 

Considering all these aspects, electrospun nanofiber mats are excellent candidates to act as a support matrix for various metal or semiconducting NPs, giving stable hybrid materials. Specifically, efficient hybrid systems consisting of photocatalytic nanoparticles supported on polymeric nanofibers can be obtained simply, incorporating semiconducting NPs onto the surface of preformed polymeric NFs or introducing the NPs in the polymer solution before the electrospinning process. Although several works have been conducted on the application of hybrid electrospun nanofiber systems to degrade toxic organic compounds in wastewater via photocatalysis, to the best of our knowledge, there is no comprehensive review that summarizes the relevant literature and provides critical insights, especially from a perspective of the role developed by the polymeric nanofibers. Consequently, with the rapid increase in this research topic, in this brief review, advances in the use of electrospun NFs based materials with photocatalytic activity in light-driven wastewater remediation processes are presented. The role played by various electrospun polymer nanofibers in the preparation of these systems and their application in the photodegradation of different types of organic pollutants is described. Finally, concluding remarks and future perspectives of this research area of technological interest are also outlined.

## 2. Electrospun Polymer Nanofibers

**Figure 1:**
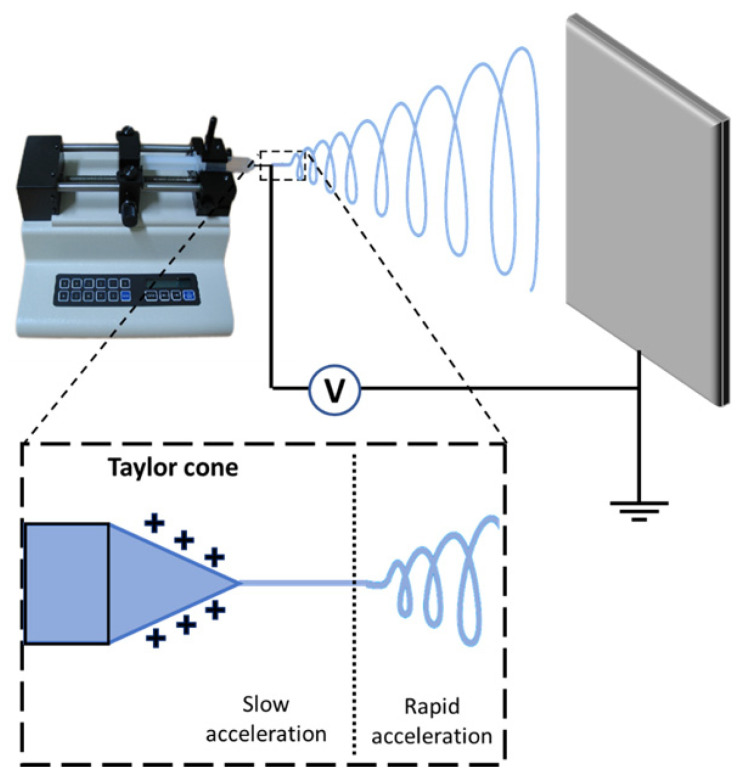
Scheme of the electrospinning setup and process.

Electrospinning is a versatile technology to produce polymer nanofibers, from solutions or melts, using electrostatic forces. Typically, an electrospinning setup is composed of a syringe, a collector, and a high voltage power supply (See Figure 1). The syringe acts as a polymer solution reservoir and has a metal capillary at the tip connected to the positive terminal of a high voltage source. The negative terminal is connected to a metal collector that, in most cases, is simply grounded. The flow of the electrospinning process is controlled by a pump that generates adequate drops at the needle tip. When the charges accumulate on the drop’s surface (due to the high voltage), mutual charge repulsion and the contraction of the surface charges to the counter electrode cause a force directly opposite to the surface tension [9]. When the intensity of the electric field increases, the surface of the fluid at the tip of the capillary tube elongates, forming a conical shape known as the Taylor cone (Figure 1). Specifically, the electrospun NFs fabrication process requires a critical voltage at which the repulsive electrostatic force overcomes the surface tension. The charged fluid jet is ejected from the tip of the Taylor cone toward the collector. Then, the polymer solution jet experiences an instability and elongation process, which allows the jet to become very long and thin. During this last process, the solvent in the jet evaporates, allowing the collection of an interconnected mat made up of ultra-thin fibers [9].

The tunable process parameters governing electrospinning are the applied voltage, the distance between the tip and the collector, the feed rates of solutions, the rotational speed of the collector, and the type of collector. At the same time, some intrinsic parameters of the polymeric solutions that affect the characteristics of the final material are surface tension, viscosity, concentration, conductivity, dielectric constants of the solvent and polymer, polymer molecular weight and polydispersity, polymer chemical structure, and presence of interactions, and electrostatic forces. Moreover, some environmental parameters, such as humidity and temperature, affect the electrospinning process [10,11,12,13,14,15,16]. Several reviews have addressed the effect of these parameters on the properties of the obtained electrospun NFs [11,12,13]. It is worth noting that the parameters governing conventional electrospinning to obtain single nanofibers are shared with coaxial electrospinning, a process aimed at obtaining core–shell type nanofibers with various morphologies from two or more different polymer solutions [14]. 

However, it should be noted that, among others, the two main parameters that strongly affect the electrospinning process are polymer concentration and electrical potential [12]. In particular, the concentration of the solution strongly affects the size of the fibers, e.g., the diameter of the electrospun NFs increases with the concentration of the solution according to a ratio of the power-law [12]. At the same time, electrospinning voltage strongly correlates with the formation of defects in the fibers [12]. 

In general, many highly soluble polymers, which give rise to sufficiently concentrated solutions, can be processed through the electrospinning process to obtain fibers. It is estimated that more than 200 different polymers and copolymers, both synthetics, and naturals, have been electrospun to obtain NFs, including the following examples, poly (lactic acid), poly (lactic-co-glycolic acid), poly (caprolactone), poly (ethylene oxide), poly (vinyl alcohol), and cellulose [17,18,19,20,21,22]. In Table 1, some examples of synthetic and natural polymers that have been electrospun, including the solvent used, are summarized [23]. Interestingly, some inorganic solids, such as metals, metal oxides/carbides/nitrides, as well as carbon and doped carbon, which have been processed as fibers using polymers as carriers, are also included in Table 1.

## 3. Hybrid Photocatalysts Based on Polymer Nanofibers in the Degradation of Water Pollutants

The organic contaminants in water originally come from domestic, municipal, and industrial wastewater, and therefore there are various organic and inorganic pollutants. Organic contaminants, such as dyes, antibiotics, analgesics, herbicides, pesticides, and stimulants, have become the main sources of water contamination. Therefore, research for developing new approaches for the effective removal of these pollutants, such as photocatalytic processes, has received increasing attention [24]. Generally, the photocatalytic capacity of a material in the degradation of organic pollutants in water is determined first using model molecules of organic pollutants. Organic dyes are the most studied molecules to test the degradation activity of many materials. Dyes are commonly used in industrial and laboratory activities, although it is recognized that they have a substantial negative impact on the environment.

Interestingly, dye degradation (usually denoted by changes of color or discoloration phenomena) can be observed even by the naked eye and easily monitored by UV-Vis absorption measurements. Some of the most widely used organic dyes are azo dyes, such as Reactive Black 5 (RB5), Reactive Red 120 (RR120), Reactive Orange 16 (RO16), among others [25,26,27,28]. Additionally, it is nowadays known that pharmaceutical molecules are not only beneficial drugs for the cure of diseases, but also are possible water contaminants. Recently, about 3000 different biologically active compounds have been reported in the water cycle, including surface water, wastewater, groundwater, and, on a smaller scale, drinking water [29].

As mentioned, polymer nanofibers have proven to be a good alternative to support photocatalyst particles due to their advantageous characteristics, such as high porosity, high specific surface area, flexibility, and the chemical functionalization possibilities that these materials present. Thus, hybrid polymeric NFs, in the form of permeable free-standing or supported layer on porous substrates, have been demonstrated to act not only as good support and stabilizer for photocatalytic NPs, but also as an active phase aiding in the degradation of pollutants, e.g., mitigating the inhibition process of oxidative capacity that the co-occurring organics commonly produce by the scavenging of ROS and electron holes during photocatalysis [30]. Indeed, NFs can improve the effectiveness of the photocatalytic water remediation process by attracting the contaminants towards the NPs or favoring the predominant transport phenomena (e.g., diffusion) of water in the photoactive material.

The following sections summarize the research found in the literature on the application of photocatalytic hybrid nanofibers in water and wastewater remediation to date. The research cases reviewed are presented grouped into four categories according to the polymeric material used to obtain the nanofibers, i.e., Section 3.1 synthetic polymers, Section 3.2 natural polymers, Section 3.3 copolymers and polymeric blends, and Section 3.4 polymers blended with carbon nanostructures.

### 3.1. Electrospun Nanofibers of Synthetic Polymers Decorated with Photocatalytic Nanoparticles

Most of the electrospun nanofibers that have been reported as supports of photocatalysts for the degradation of organic water pollutants are formed by synthetic polymers. To obtain the hybrid material, photocatalytic nanoparticles can be either mixed with the polymer before electrospinning or deposited on the polymer nanofibers after electrospinning. In this section, the use of different synthetic polymers to obtain hybrid polymer nanofibers/photocatalytic nanoparticles is featured, especially highlighting the contribution made by the polymer in the photocatalytic performance of the material. 

**Figure 2:**
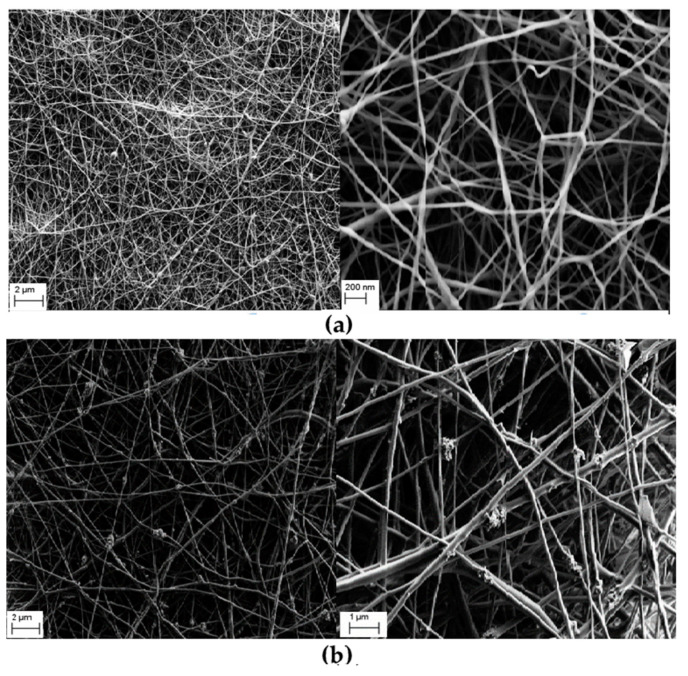
SEM images of (**a**) PA6 NFs and (**b**) NFs of PA6 with 25 wt.% TiO_2_ nanoparticles. (Reprinted from the work of Blanco et al. [25]).

A representative work was developed by Blanco and coworkers, who prepared electrospun polyamide 6 (PA6, a thermoplastic polymer with good mechanical properties) nanofibrous membranes containing TiO_2_ nanoparticles [25]. The authors incorporated the nanoparticles into the polymer solution before the electrospinning process. Thus, to prepare the hybrids NFs, 25 wt.% TiO_2_ NPs were added to a PA6 solution (12 *w*/*w* in a mixture 2:1 of acetic acid: formic acid) using a conventional ultrasonic bath and sodium dodecyl sulfate to stabilize the NPs dispersion. The electrospun nanofibrous membranes were obtained at 23 ± 2 °C and 50 ± 5% of relative humidity; the collector distance and voltage were set at 170 mm and 75 kV, respectively. The authors estimated that most of the NPs were inside the PA6 NFs, which is based on the significant increase in the diameters of the NFs ranging from 60–100 nm for bare PA6 NFs to 110–260 nm for PA6/25 wt.% TiO_2_ NFs. The FTIR characterization corroborated the presence of the TiO_2_ NPs, showing an increase in the band centered at 550 cm^−1^, which was attributed to Ti–O bending. However, the FTIR spectrum of NFs modified with TiO_2_ NPs did not show significant changes that could reflect interactions between PA6 and TiO_2_ compared to the spectrum of non-modified nanofibers. Therefore, if there are interactions, these would not be strong enough to be detected by FTIR spectroscopy. The absence of strong interactions between TiO_2_ NPs and PA6 was also demonstrated by the TiO_2_ NPs agglomeration in the PA6/25% TiO_2_ NFs observed by SEM images in Figure 2. In the case of photodegradation study, 80% degradation of Remazol Black B, a synthetic diazo reactive dye used as a model of organic water pollutants, was observed in a solution with the hybrid nanofibrous membranes immersed after 240 min of irradiation with 365 nm UV light. This photocatalytic process was monitored following the decrease in the dye absorbance using UV-Vis spectroscopy. Notably, the photocatalytic hybrid nanofibers maintain their effectiveness when reused for three cycles. Additionally, the authors also studied the antibacterial activity of PA6/TiO_2_ electrospun NFs against *E. coli*, which was eliminated after 24 h of contact with the material upon UV light irradiation. 

**Figure 3:**
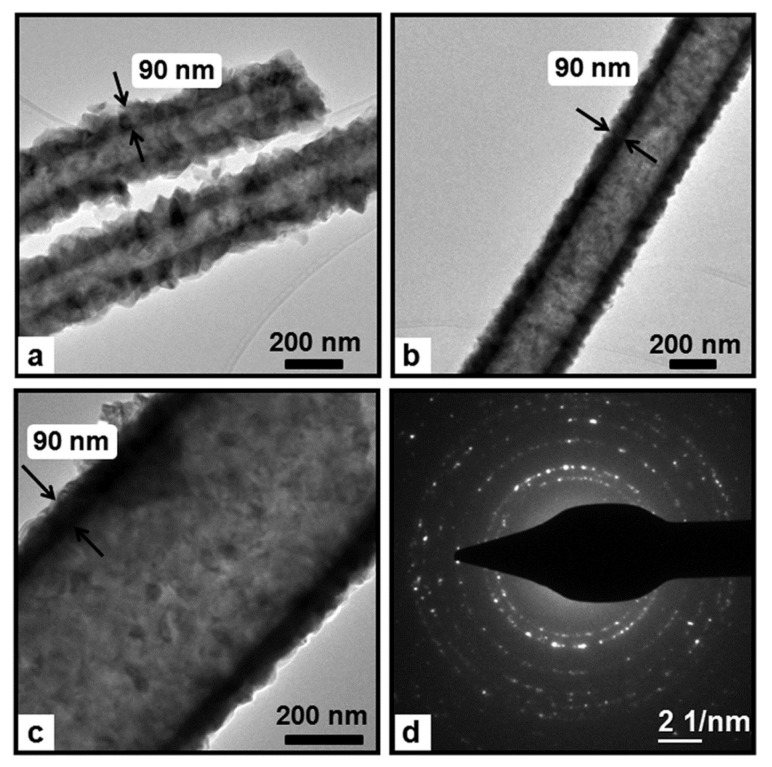
Representative TEM images of (**a**) 8%-nylon 6,6/FA-ZnO, (**b**) 5%-nylon 6,6/HFIP-ZnO, (**c**) 8%-nylon 6,6/HFIP-ZnO core−shell nanofibers; (**d**) representative SAED pattern of the core−shell nylon 6,6-ZnO nanofibers (8%-nylon 6,6/HFIP-ZnO NF). (Reprinted from the work of Kayaci et al. [31]. Copyright 2012, with permission from the American Chemical Society).

Well-documented homopolymer Nylon 6,6, a polyamide commonly obtained the by polycondensation of hexamethylenediamine and adipic acid, has also been used to prepare polymer-inorganic core–shell NFs, with a polymeric core and an inorganic shell of zinc oxide, combining electrospinning and atomic layer deposition (ALD) techniques [31]. Nanofibers of Nylon 6,6 were obtained by electrospinning using 5 wt.% and 8 wt.% polymer concentrations and two different solvents, formic acid (FA) and hexafluoro-2-propanol (HFIP). The electrospinning was carried out at 23 °C and 36% relative humidity, and syringes with metallic needles of 0.8 mm of inner diameter were used, while a feed rate of 1 mL/h, 15 kV of applied voltage, and a tip-to-collector distance of 10 cm were set up. Later, ZnO with precise thickness control was deposited onto NFs using the ALD technique. The average diameter of the Nylon 6,6 NFs showed dependence on both the concentration of the electrospun solution and the solvent used. However, the TEM images revealed that the thickness of the ZnO shell layer was about 90 nm for each Nylon 6,6-ZnO nanofiber sample, independently of the average fiber diameters of the bare polymer previous to performing the ALD technique (Figure 3). Remarkably, the fibrous structure of Nylon 6,6 was not disturbed during the ALD of ZnO shell layer, a process developed at 200 °C. The core−shell nylon 6,6-ZnO nanofibers showed unique properties, such as structural flexibility, provided by the polymeric core and good photocatalytic activity due to the ZnO shell layer. The photocatalytic activity of the nanofibers obtained was tested by monitoring the photocatalytic decomposition of Rhodamine-B, an organic dye molecule used as a model of organic waste compound. The Nylon 6,6-ZnO nanofiber mat of thinner average fiber diameter (~80 nm) showed better photocatalytic efficiency (93% RhB degradation after 16 h of 365 nm UV irradiation) when compared to the nanofiber mat having AFD of ~650 nm, likely due to the higher surface area of this sample. The structural and chemical stabilities of the Nylon 6,6-ZnO nanofiber mats were tested by TEM imaging and XPS measurement after the UV irradiation experiment. TEM images showed that the samples maintained their nanofibrous structure without deformation, only a few spots due to the destruction of ZnO layer were detected, and the XPS study of the samples corroborated these observations. 

**Figure 4:**
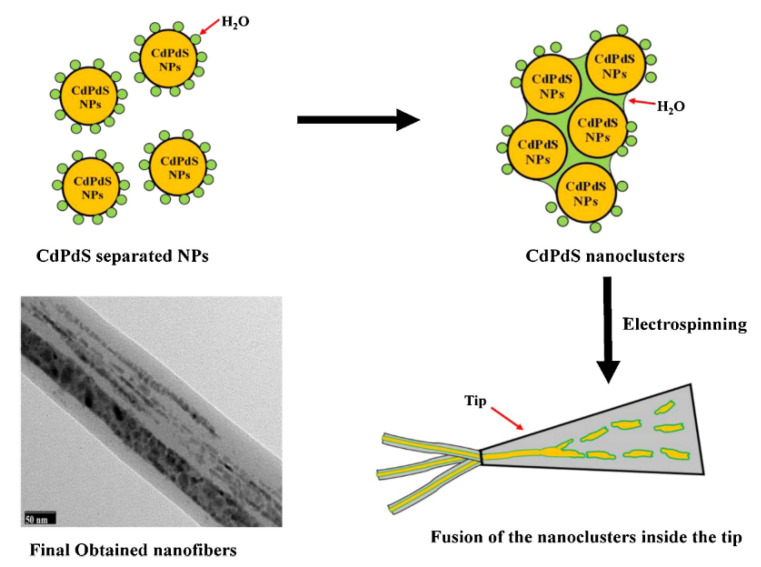
Illustration showing the mechanism of forming the core–shell structure. (Reprinted from the work of Unnithan et al. [27]; Copyright 2012, with permission from Elsevier).

Unnithan and coworkers incorporated photocatalytic nanoparticles ex situ to the electrospinning process to produce hybrid NFs. They report the preparation of cadmium palladium sulfide (CdS/PdS) alloy NPs embedded in the NFs of thermoplastic poly(vinyl acetate) (PVAc), known for its adhesion properties [27]. In this case, the synthesis of the semiconducting photocatalytic nanoparticles was developed in situ in the polymer solution. The in situ synthesis of CdS/PdS alloy NPs was carried out by mixing ammonium sulfide with the respective salt precursors of Cd and Pd in a PVAc DMF solution [27]. The electrospinning of the colloidal solution thus obtained produced nanofibers of core–shell structure. NFs with uniform diameters in the range of 200–250 nm were obtained. The core–shell configuration of NFs was evidenced by TEM and HRTEM imaging (Figure 4). To explain the results, the authors suggested that the water molecules from the reagents promoted the assembly of the NPs to form initially nanoparticle clusters and, subsequently, the NPs clusters would coalesce and form the core section of the NFs, facilitated by the hydrophobic nature of the polymer. The core–shell NFs were tested against two different azo dyes, RB5 and RO16, showing almost 100% of dye degradation after 180 and 120 min under sunlight irradiation, respectively. Notably, the efficiency was not affected after three consecutive cycles. The PVAc shell did not influence the photocatalytic activity of the PdS/CdS NPs. The authors attribute this to the suitable electric conductivity of the PVAc. This polymer would form a conductive network on the surface of NPs, which can promote the photogeneration of electron (e−) and holes (h+) and subsequently prevent their recombination.

**Figure 5:**
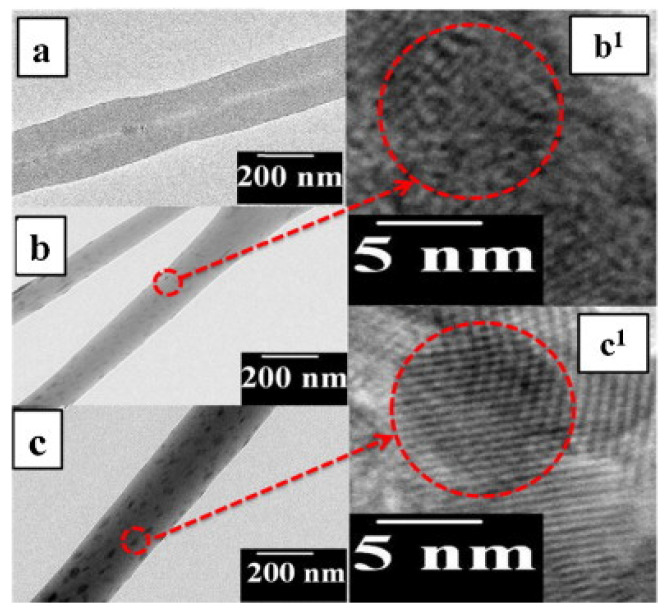
TEM images of (**a**) pristine PVAc electrospun nanofibers, (**b**) ZnS–PVAc hybrid electrospun nanofibers, (**c**) PdSZnS–PVAc hybrid electrospun nanofibers, and (**b^1^**,**c^1^**) HRTEM for the marked area in (**b**,**c**) hybrid electrospun nanofiber mats, respectively. (Reprinted from the work of Panthi et al. [32], Copyright 2015, with permission from Elsevier).

Panthi and coworkers reported a similar photocatalytic system; they prepared hybrid electrospun NFs of PVAc containing PdS/ZnS NPs [32]. The NPs were synthesized in situ by the following summarized procedure. ZnAc and PdAc with 5:1 weight ratio were dissolved in DMF. The prepared solution was mixed with 18% (*w*/*w*) PVAc solution in DMF. The resulting solution was kept under stirring for 2 h to ensure proper mixing, and then ammonium sulfide was added dropwise with vigorous stirring for 5 h to promote the fine dispersion of PdS-doped ZnS NPs. Finally, the polymer and PdS/ZnS NPs solution was electrospun. Hybrid NFs with smooth surfaces and uniform diameters in the range of 200–300 nm were obtained. The absence of PdS and ZnS NPs on the surface of the hybrid nanofibers was confirmed by FESEM images, which indicated that the PdS and ZnS NPs were incorporated into the polymer nanofibers. In Figure 5, TEM and HRTEM images of pristine PVAc and hybrid ZnS-PVAc and PdSZnS-PVAc electrospun nanofibers are shown. The images of hybrid electrospun NFs show well-doped nanoparticles inside the fiber matrix. In both cases, the size of the nematic phase constituted of NPs was in the range of 5–8 nm. The authors propose that the position of metal ions (M^2+^) on electrospun nanofibers should be adjusted by controlling the interaction between the polymer and metal ions. During the electrospinning process, Coulomb repulsions between the charged metal nanoparticles (M^2+^) could be the main factor responsible for the NPs homogeneously dispersed, favoring polymer/metal ion interactions. Remarkably, the photocatalytic activity of PdSZnS–PVAc hybrid nanofibers was higher than that of ZnS–PVAc hybrid nanofibers. The complete degradation of the MB dye was achieved with PdS/ZnS-PVAc in 100 min, while only 75% of the dye was degraded simultaneously with ZnS–PVAc. 

**Figure 6:**
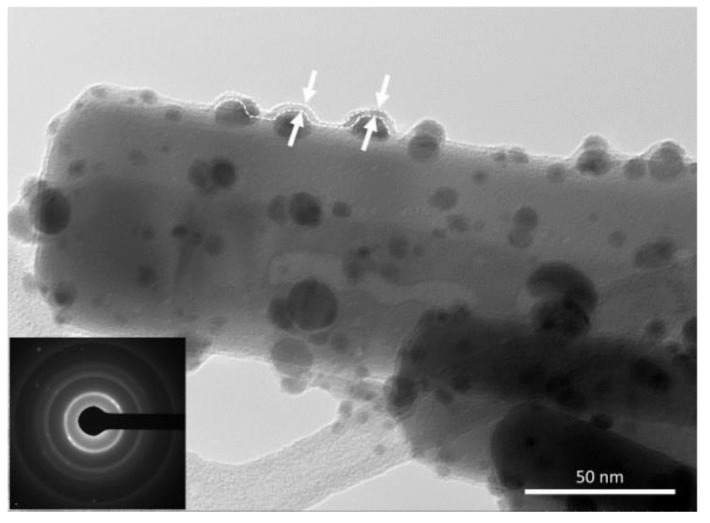
TEM images of PVDF/ZnO/Ag_2_CO_3_/Ag_2_O electrospun nanofiber; inset SAED pattern. (Reprinted from the work of Rosman et al. [28]).

Rosman and coworkers used the highly chemically inert thermoplastic fluoropolymer polyvinylidene fluoride PVDF to obtain a series of electrospun NFs with embedded NPS of the heterojunction photocatalysts of ZnO with different Ag-based compounds [28]. To prepare the NFs, first, a polymer solution of PVDF in DMAc/acetone blend, was doped with different photocatalysts (ZnO, ZnO/Ag_2_CO_3_, ZnO/Ag_2_CO_3_/Ag_2_O, and ZnO/Ag_2_O) by stirring, and then the resulting doped solution was used to fabricate electrospun NFs via electrospinning. The set parameters were: flux 1 mL/h, a voltage of 8 kV, and a distance of 150 mm between the spinneret and the collector. The NFs showed a rough surface, and the ZnO/Ag_2_CO_3_/Ag_2_O nanoparticles were homogeneously distributed in PVDF NFs (Figure 6). The FTIR spectra of the nanofibers with the embedded heterojunction photocatalysts ZnO/Ag_2_CO_3_, ZnO/Ag_2_O, or ZnO/Ag_2_CO_3_/Ag_2_O demonstrated the structural integrity of the photocatalysts. Significantly, the main signals of ZnO, Ag_2_CO_3_, and Ag_2_O were not overlapped after being incorporated in the PVDF nanofiber. The NFs with the ZnO/Ag_2_CO_3_/Ag_2_O photocatalyst showed faster degradation of Reactive Red 120 (RR120) dye as a model pollutant under 312 nm UV light irradiation. In fact, 99.62% dye degradation when 300 min of light exposure was reached, maintaining a relatively high photocatalytic activity after five cycles. This good activity was attributed to a synergistic effect of adsorption and enhanced photocatalytic degradation. According to contact angle and AFM measurements, the system catalyst embedded into the PVDF NFs would exhibit lower hydrophobization and higher surface roughness, which would facilitate the dye molecule adsorption adjacent to their photocatalytic surface sites. On the other hand, the heterojunction between the ZnO and Ag_2_CO_3_/Ag_2_O mixed phase would narrow the bandgap. The authors highlight the affinity of PVDF and RR120, indicating that bare PVDF nanofibers showed adsorption of approximately 10% of dye after 300 min of UV light irradiation. 

Ramasundaram and coworkers reported the fabrication of hybrid NFs PVDF–photocatalytic nanoparticles, using different photocatalytic NPs [33]. In their work, TiO_2_ NPs were incorporated by electrospray on electrospun NFs of PVDF, i.e., the NP incorporation was made after the electrospinning process. First, the PVDF NFs mat was prepared by the electrospinning process using a PVDF solution in a DMF/acetone mixture. The electrospinning setup used comprised a needle with an inner diameter of 330 μm, a spinning speed of 50 μL/min, 10 cm distance between the tip and the collector, and 15 kV of applied voltage. Then, a TiO_2_ dispersion in DMF was electrosprayed on both sides of the PVDF NFs mats using the same process parameters used for PVDF electrospinning. According to the authors, DMF is one of the best solvents for PVDF based on solubility parameter theory. Thus, at room temperatures, DMF could penetrate the amorphous regions of PVDF, while the crystalline regions remain mostly unaffected, producing minimal swelling. This effect of the solvent on the polymer would allow to immobilize electrosprayed nanomaterials efficiently on the surface of the PVDF matrix. The resultant PVDF–TiO_2_ hybrid NFs presented TiO_2_ NPs mainly immobilized on the outer surface of the mat. Notably, the hybrid PVDF–TiO_2_ NFs completely degraded cimetidine, bisphenol A (BPA), and 4-chlorophenol upon 40, 80, and 100 min of UV irradiation, respectively. The photocatalytic activity of the PVDF–TiO_2_ was observed during 10 cycles of catalysis; prior to each cycle, the photocatalyst was rinsed with deionized water and immersed in a freshly prepared target solution before new UV irradiation. Interestingly, the authors report no noticeable reduction in photocatalytic activity after the 10 cycles, indicating the high reusability of the hybrid system. Additionally, no trace of Ti into the treated aqueous solutions was detected, confirming the robustness of the PVDF–TiO_2_ hybrids NFs, a product of the above-mentioned DMF effect on the polymer and the interactions between PVDF and TiO_2_ nanoparticles.

**Figure 7:**
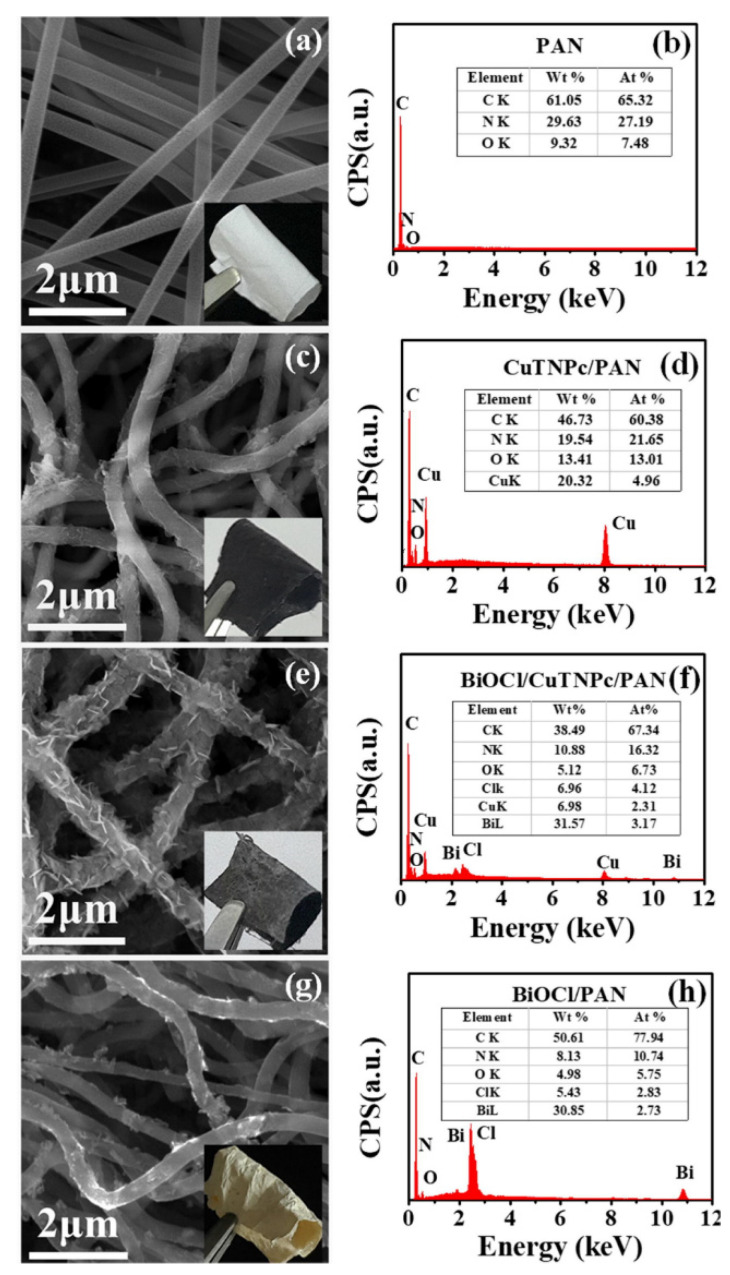
SEM images and EDX spectra of samples: (**a**,**b**) PAN, (**c**,**d**) CuTNPc/PAN, (**e**,**f**) BiOCl/CuTNPc/PAN, and (**g**,**h**) BiOCl/PAN. (Reprinted from the work of Guo et al. [34]. Copyright 2018, with permission from Elsevier).

Another synthetic polymer that has been frequently used in the production of synthetic nanofibers to immobilize photocatalytic nanostructures is polyacrylonitrile (PAN), a vinyl polymer derived from the acrylate family. For example, Guo and coworkers reported the use of PAN to immobilize nanostructures of 2,9,16,23-tetranitro phthalocyanine copper (II) (CuTNPc) and bismuth oxychloride nanosheets (BiOCl) [34]. PAN NFs were obtained by simple electrospinning of a solution in DMF, using 12 kV of operating voltage and 15 cm of distance between the syringe tip and the collector. Scanning electron microscopy (SEM) images of the obtained PAN NFs showed ultra-long one-dimensional nanostructures, with a high aspect ratio and a relatively smooth surface, their diameter distribution ranging from 200 to 300 nm. Two consecutive solvothermal treatments modified PAN NFs. Firstly, the NFs were treated at 160 °C for 12 h with a 4-nitrophthalonitrile, Cu(Ac)_2_H_2_O, and ammonium molybdate in ethylene glycol solution to form CuTNPc/PAN NFs. Secondly, CuTNPc/PAN NFs were reacted with Bi(NO_3_)_3_ 5H_2_O and NaCl dissolved in ethylene glycol at the same temperature for 24 h to obtain BiOCl/CuTNPc/PAN NFs. Note that both processes were carried out in a 25 mL Teflon-lined stainless-steel autoclave. After the first solvothermal treatment, the PAN NFs surface was covered with a secondary nanostructure of CuTNPc, maintaining the non-woven nanofiber structure. In this case, a noticeable color change from white to black was observed. After secondary solvothermal reaction, the BiOCl nanosheets were uniformly grown on the surface of CuTNPc/PAN nanofibers without aggregation. Thus, the three-dimensional macroporous structure was well maintained. The BiOCl/PAN nanofibers were also prepared similarly. Figure 7 shows typical SEM images of PAN, CuTNPc/PAN, BiOCl/CuTNPc/PAN, and BiOCl/PAN nanofibers mats, as well as their respective energy-dispersive X-ray (EDX) spectra.

The analysis of the thermogravimetric profiles of pristine PAN NFs and modified NFs (CuTNPc/PAN, BiOCl/CuTNPc/PAN, and BiOCl/PAN) revealed a decrease in the thermal stability of the modified NFs compared to those of bare PAN. This allowed inferring the existence of interactions between PAN and metal atoms, explaining the uniform distribution of heterostructures in NFs and the absence of aggregation observed. Concerning their photocatalytic activities, BiOCl/CuTNPc/PAN NFs showed 75% RhB degradation after 180 min under UV irradiation, while for CuTNPc/PAN nanofibers and BiOCl/PAN nanofibers, the values were only 24% and 20%, respectively. Interestingly, the authors reported an adsorption of approximately 5% of RhB by PAN NFs, allowing to infer that an additional effect produced by PAN could exist, e.g., favoring the diffusion of the dye toward the photoactive sites on the NFs. Note that the flexible nanofibrous mat easily floated on the surface of the liquid, optimizing the absorption of sunlight during the photocatalysis step.

**Figure 8:**
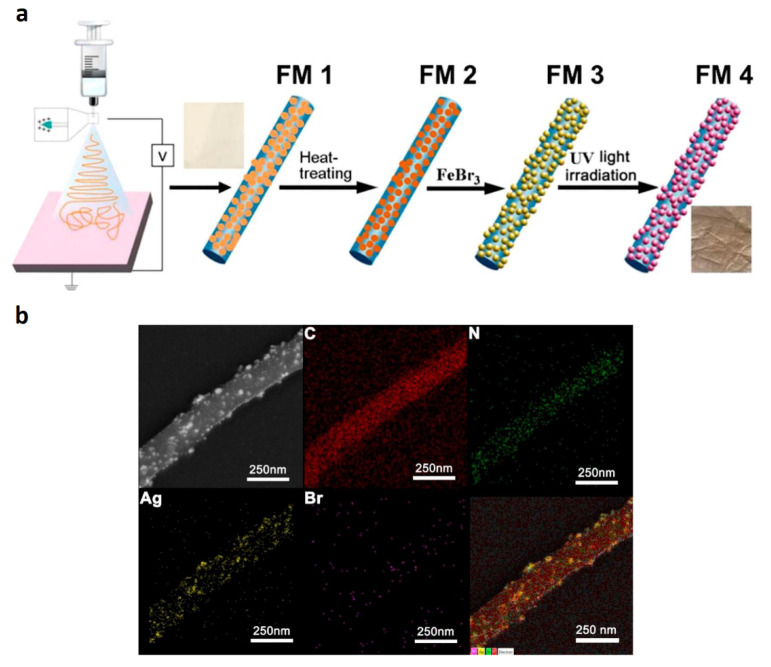
(**a**) Schematic diagram for the fabrication procedure of PAN/AgBr/Ag fibrous membrane and (**b**) STEM image and corresponding EDX elemental mapping of PAN/AgBr/Ag nanofibrous membrane. (Adapted from the work of Qayum et al. [35], Copyright 2019, with permission from Elsevier).

On the other hand, Qayum and coworkers proposed an attractive strategy to promote the photocatalytic degradation of the widely used salicylic acid (SA), using PAN nanofibers as support for photocatalytic nanoparticles [35]. In this study, a combination of electrospinning, wet-chemical methods, and thermal treatment to obtain hydrophilic PAN/AgBr/Ag fibrous membranes was employed. The fabrication procedure involved four steps: (i) firstly, a PAN-AgNO_3_ NFs mat was prepared by electrospinning a sol of AgNO_3_ and PAN into DMF. The set electrospinning parameters were a needle with a diameter of 0.7 mm, distance of 20.0 cm between the needle and collector, 18 kV of voltage, and feeding rate of 0.1 mL/min; (ii) secondly, thermal treatment of the mat at 140 °C, to promote the transformation from AgNO_3_ to Ag nanoparticles, was performed; (iii) the treated membrane was immersed in FeBr_3_ solution for 40 min for the dissolution of AgNO_3_ and formation of AgBr nanoparticles; (iv), and finally, UV irradiation was applied to generate the AgBr/Ag Schottky junction. The procedure is summarized in the scheme of Figure 8a. Notably, according to the described procedure, the precursor PAN/AgNO_3_ nanofibers were subjected to three considerable modifications to generate the final PAN-AgBr/Ag nanofibrous material.

SEM images and energy disperse X-ray spectrometry (EDX) measurements of PAN/AgBr/Ag fibrous membranes are depicted in Figure 8b. The images show that C and N were uniformly distributed on the surface of the fibers, while Ag and Br were preferentially concentrated on the surface of NPs, demonstrating the deposition of the AgBr and Ag NPs in the outer layer of the fiber. PAN/AgBr/Ag fibrous membranes showed good stability of the porous structures along with adequate mechanical strength, even allowing their use in filtration processes. This material could degrade 96% of SA upon 5 h of UV-visible irradiation, even after five cycles of use. Interestingly, the antibacterial activity of the nanocomposite fibers was also demonstrated; the performance of the material was tested using a wastewater sample containing SA, *E. coli*, and dispersed red 1 (an azo dye).

### 3.2. Electrospun Nanofibers of Natural, Biobased and/or Biodegradable Polymers Decorated with Photocatalytic Nanoparticles

The use of natural and biodegradable polymers for various purposes, including the production of hybrid nanofibers containing photocatalytic NPs, has inherent environmental advantages over synthetic polymers, the latter being obtained from fossil-based raw materials and contributing to the increasingly dramatic and unpopular plastic pollution. Additionally, natural, biobased and/or biodegradable polymers might have chemical functionalities that favor the generation of favorable interactions with photocatalytic nanoparticles and organic pollutants. Thus, the polymer-NPs interactions contribute to the stability of the hybrid NFs, while the polymer-organic pollutant interactions facilitate the contact of the organic molecules with the active sites of photocatalysis, favoring their photodegradation. However, the studies reported in the literature using these types of polymers are still scarcely addressed. This section summarizes some reports on the use of natural, biobased and/or biodegradable polymers, such as chitosan, cellulose, polycaprolactone, and polylactide, to prepare hybrid photocatalytic nanofibers.

Park and coworkers successfully prepared electrospun nanofibrous mats of biodegradable, biocompatible, and hydrophobic polyester polycaprolactone (PCL) containing TiO_2_ NPs [26]. In their study, they prepared PCL NFs with TiO_2_ NPs at various concentrations (1, 3, 5, and 7 wt.%). The electrospinning solutions were prepared from a pre-dispersion of the TiO_2_ powders in a methylene chloride/DMF polymer solution that was stirred for 5 h and then blended with a 12.5 wt.% PCL solution for 6 h. The set electrospinning parameters were: tip-to-collector distance 20 cm, capillary tip with 0.51 mm of inner diameter, and a voltage of 18–21 kV. Subsequently, the NFs were treated by atmospheric pressure plasma with oxygen gas to decompose the polymer at the surface and expose TiO_2_ at the exterior of nanofibers. The treatment with high-energy plasma etched the polymer, and as a consequence, the exposing of TiO_2_ clusters at the surface of PCL NFs increased the number of active sites of TiO_2_. The results showed that a concentration of TiO_2_ up to 3 wt.% had a reinforcing effect on the polymeric membranes. Conversely, above 5 wt.% TiO_2_, the tensile strength decreased probably because of the severe agglomeration of the TiO_2_ nanopowder that was observed in SEM images. The material showed 70–90% RB5 degradation (3 mg/mL solution) after 120 min of 254 nm of UV irradiation, the degradation increasing with the increase in the amount of TiO_2_ NPs. The material with 3 wt.% TiO_2_ NPs showed the best compromise between mechanical strength and improved photocatalytic capacity. The photocatalytic activity of these systems was explained by two concomitant effects resulting from the plasma treatment: the exposure of the NPs at the fiber surface due to polymer etching and a high increase in fiber hydrophilicity evidenced by contact angle measurements. The authors suggested that, even if the TiO_2_ NPs played a role in increasing in part the hydrophilicity of the nanofibers, the most important factor would be the generation of polar OH groups at the polymer surface by atmospheric pressure plasma treatment, corroborated by FTIR. This would allow the water to spread completely over the surface and increase the effective number of photocatalytic active sites on the large surface [26]. Thus, the authors demonstrated that a plasma treatment can efficiently modify PCL/TiO_2_ hybrid nanofibers from a hydrophobic to a hydrophilic state.

**Figure 9:**
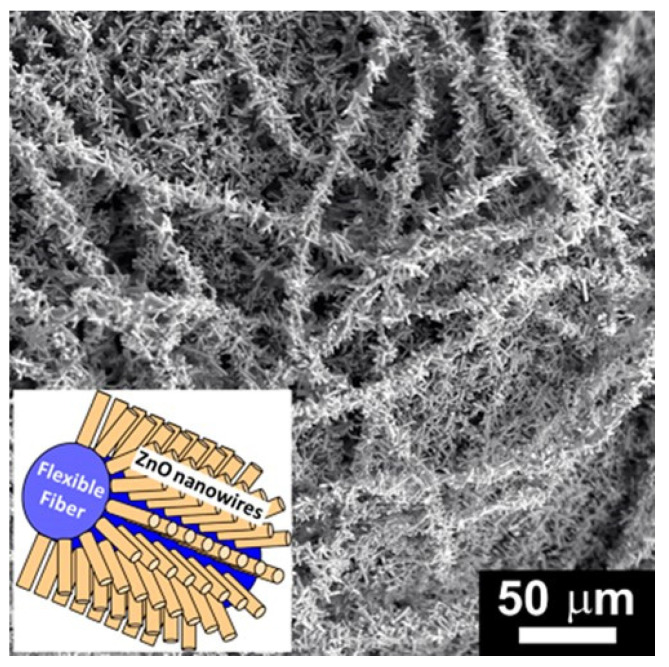
Hierarchical fibrous polymer/ZnO nanostructures. Low-magnification SEM image of the hierarchical nanostructure consisting of radially aligned ZnO nanowires grown on electrospun poly-L-lactide nanofibers. Inset: schematic representation of the hierarchical nanostructure. (Reprinted from the work of Sugunan et al. [36]. Copyright 2021, with permission from John Wiley and Sons).

Another biodegradable and biobased thermoplastic polyester, Poly-L-Lactide (PLLA), was used by Sugunan and coworkers [36] to prepare a highly flexible hybrid material consisting of PLLA NFs with radially oriented ZnO nanoneedles. To obtain the NFs, the electrospinning of a 7 wt.% PLLA/chloroform solution was carried out. In the process, a 0.8 mm diameter needle, a voltage of 10 kV, 10 cm distance from the tip of the needle to the collector and a flow rate of 1 mL/h were used. Later, PLLA NFs were immersed for 30 min into a colloidal ZnO suspension and then allowed to dry. Then, the “seeded” NFs were immersed into a mixed aqueous solution of Zn(NO_3_)_2_ and hexamine and heated to 75 °C for 6 h. The NFs obtained in this way showed a hierarchical three-dimensional nanostructure consisting of polymeric NFs (~200 nm before deposition) that were densely covered by radially oriented ZnO nanoneedles (~50 nm), as Figure 9 shows. The authors determined that a typical 10 × 10 cm mat had a surface area equal to a square of Si/glass substrate of 75 cm side length through BET surface area measurements.

Interestingly, the hierarchical PLLA-ZnO nanostructure retains the high flexibility of the original PLLA nanofibrous mat. This last allowed the authors to design a water treatment by setting up a “continuous flow” through fixed-bed photocatalytic material placed inside a glass tube and using a 100 W mercury vapor lamp as a UV light source. A 90% of methylene blue decomposition was obtained after 80 min of circulation of a 5 ppm solution in the system, while at least a 50% of degradation of two hazardous compounds, MCP an organophosphate plaguicide and diphenylamine were achieved after 100 min of irradiation. Additionally, the material exhibited the high structural and chemical stability of the hierarchical structures in the continuous flow regime.

Chitosan, a non-toxic, biodegradable, and environmentally friendly biopolymer, is considered a promising material for several applications, e.g., as a carrier due to its hydroxyl and amino groups, which allow it to interact with various molecules and nanoparticles. Accordingly, chitosan nanofibers are highly interesting for the generation of hybrid materials; however, the electrospinning of chitosan presents considerable difficulty due to its polyelectrolyte nature and problems generated by their charges in the electrospinning process, such as instabilities in the electrospinning jet, high electrical conductivity, and viscosity of polyelectrolyte solutions (at the concentrations suitable for electrospinning) [37].

**Figure 10:**
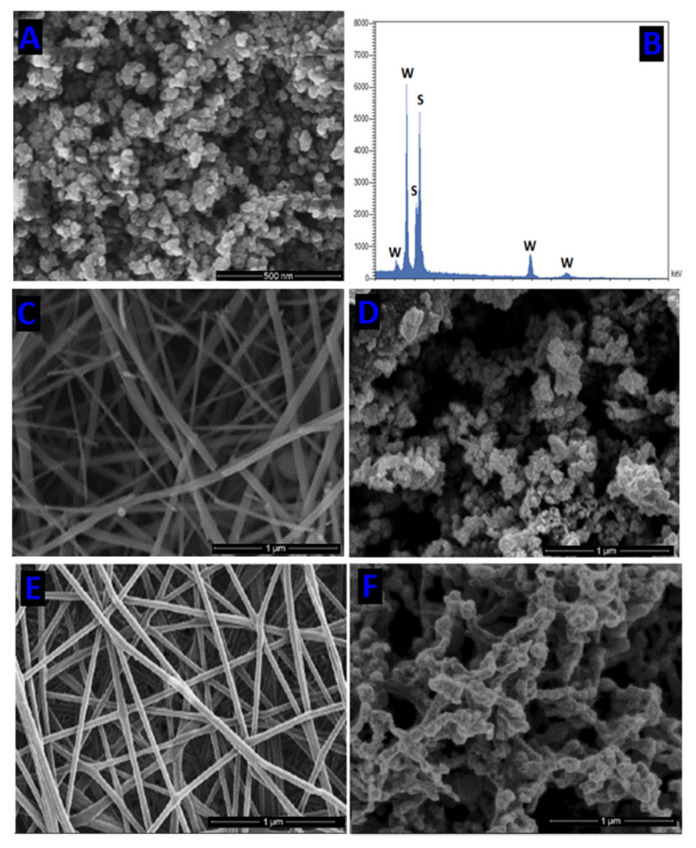
SEM image and EDS of WS2 nanoparticles (**A**,**B**), SEM image of chitosan nanofibers (**C**), WS2/chitosan nanocomposites (**D**), polycaprolactone nanofibers (**E**), and WS2/polycaprolactone nanocomposites (**F**). (Reprinted from the work of Fakhri et al. [38]. Copyright 2018, with permission from Elsevier).

Consequently, the electrospinning of chitosan remains a challenge not completely solved at present. Fakhri and coworkers prepared chitosan and polycaprolactone biodegradable polymer nanofibers decorated with Tungsten disulfide (WS_2_) and studied these hybrid nanocomposites in the photocatalytic degradation of Neomycin (NEO), an aminoglycoside antibiotic [38]. The chitosan NFs were obtained by electrospinning of low molecular weight chitosan in trifluoroacetic acid solution; the nanofiber neutralization was performed in an aqueous solution of K_2_CO_3_ and a final drying at 60 °C. The electrospinning setting was a flow rate of 1 mL/h, voltage of 13–15 kV, needle diameter of approximately 0.1 mm, 25 °C, and 50–60% relative humidity. A similar procedure was used to obtain PCL (80,000 g/mol) NFs. The WS_2_ nanoparticles immobilized on CS and PCL nanofibers were synthesized in situ by the following procedure. First, the CS and PCL nanofibers were immersed in a suspension of 0.005 M (NH_4_)_6_W_7_O_24_·4H_2_O, 0.02 g PVP, and C_2_H_5_NS (0.04 M). Secondly, an aqueous solution of acetic acid (40 mL, 5 % vol) was added under vigorous stirring. Finally, the mixture was placed in a Teflon autoclave and sealed at 140–190 °C for 8 h. Figure 10 shows SEM images and the energy dispersive spectroscopy (EDS) analysis of WS_2_ NPs and SEM images of chitosan and PCL NFs, WS_2_/chitosan and WS_2_/PCL nanocomposites. The degradation efficiencies of NEO by the photocatalytic activity of WS_2_/CS nanofibers and WS_2_/PCL nanofibers were determined following the change in characteristic absorbance of NEO solutions by UV-Vis spectroscopy. The optimal time of UV-light irradiation and pH values for high efficiency of NEO decomposition (between 80–90%) were pH 7, and 40 min. Interestingly the WS_2_/chitosan and WS_2_/PCL NFs showed higher NEO photodegradation than the WS_2_ NPs, indicating an enhancement by including polymers. 

The preparation of a hybrid photocatalyst based on cellulose acetate nanofibers, the most important synthetic cellulose ester, using TiO_2_-doped chitosan microspheres and the study of its performance in the photocatalytic degradation of methyl orange (MO) in water solution was reported by Xuejuan Shi and al. [39]. In a first step, the authors obtained cellulose acetate NFs by electrospinning of a 10 wt.% CA (*w*/*w*) in an acetone/deionized water (85/15, *w*/*w*) solution. The electrospinning conditions included an applied voltage of 20 kV, 15 cm of distance between the tip of the needle and the collector, and 1.0 mL/h flow rate. Later, chitosan microspheres doped with TiO_2_ nanoparticles were embedded onto cellulose acetate nanofibers by electrospraying. A 2 wt.% chitosan (*w*/*w*) dissolved in 90% acetic acid (*v*/*v*) solution and containing different amounts of TiO_2_ nanoparticles (from 1%, to 5%, *w*/*w*) was used to electrospray. The results showed that the diameter of TiO_2_-doped chitosan microspheres lightly increased with the increase in TiO_2_ content from 1 wt.% to 3 wt.%, while that at higher content of TiO_2_ (4 and 5%), the NPs experimented agglomeration. The BET method was employed to estimate the specific surface area of nanofibrous material. For bare chitosan and chitosan–TiO_2_ spheres embedded on CA NFs, the values corresponded to 301.30 and 342.10 m^2^/g, respectively. These results reflected an increase due to TiO_2_ NPs inclusion. The removal of MO in the presence of chitosan–TiO_2_ spheres embedded in CA NFs catalyzed by visible light showed an increase up to 3 wt.% of TiO_2_ NPs on chitosan spheres and decreased when the content of TiO_2_ increased to 4 and 5 wt.%. The authors attributed this behavior to the reduction in photocatalytic reaction sites due to TiO_2_ agglomeration. It is necessary to emphasize that the MO removal capacity was not only due to photocatalytic reactions, but also due to the adsorption of the dye mainly propitiated by chitosan, which was inferred from the FTIR and XPS results. Furthermore, recycling experiments showed that fiber membranes had excellent chemical stability and reusability.

Cellulose acetate (CA) has also been used to obtain electrospun fibers decorated with photocatalytic NPs. High surface area photocatalytic cellulosic fibers containing TiO_2_ NPs were prepared by Bedford and coworkers [40] by first electrospinning solutions of CA into non-woven fiber meshes. The electrospinning experiments were performed under ambient conditions with a voltage 25 kV, flow rate of 0.5–0.6 mL/h, and 11 cm distance between spinneret and collector. Interestingly, the obtained CA nanofibers were converted to succinylated cellulose fibers through a deacetylation using KOH in ethanol and the corresponding neutralization followed by a succinylation of the hydroxyl groups using a succinic anhydride in DMF solution. Finally, the nanofibers were loaded with dispersions of 1% titania nanoparticles, previously homogenized by ultrasound, at different pH for one hour at 75 °C, and then rinsed with distilled water under ultrasound to remove unbound titania. The electrospun fiber diameter and BET surface area were varied by controlling the concentration of acetic acid in the electrospinning solution. A higher fiber diameter at a higher concentration of acetic acid was obtained. Consequently, the BET surface area showed an inverse variation, i.e., a higher surface area at minor fiber diameter, therefore at a lower acetic acid concentration. The conversion of the CA fibers to cellulose fibers and then to succinylated cellulose fibers produced a slight change in average diameter and BET surface area of the fibers. Carboxylic acid functionality was generated because these groups are known to bind titania better than acetyl and hydroxyl groups found in the CA and cellulose fibers. When identical concentrations of P25 TiO_2_ NPs were loaded onto cellulose fibers and succinylated cellulose fibers, a clear difference in the amount of titania loaded onto the carboxylic acid functionalized fibers respect FE-SEM images observed the hydroxyl containing cellulosic fibers. The TiO_2_ doped nanofibers were tested to degrade toxin microcystin-LR (MC-LR), one of the most common toxins generated by cyanobacterial algal blooms that frequently occur in various water sources around the world. The photocatalytic degradation of MC-LR by photocatalytic cellulosic fibers loaded with titania under visible and solar light was reached. The results indicated that a large surface coverage of titania and an active surface area of fiber mat are highly desirable conditions for effective MC-LR degradation.

**Figure 11:**
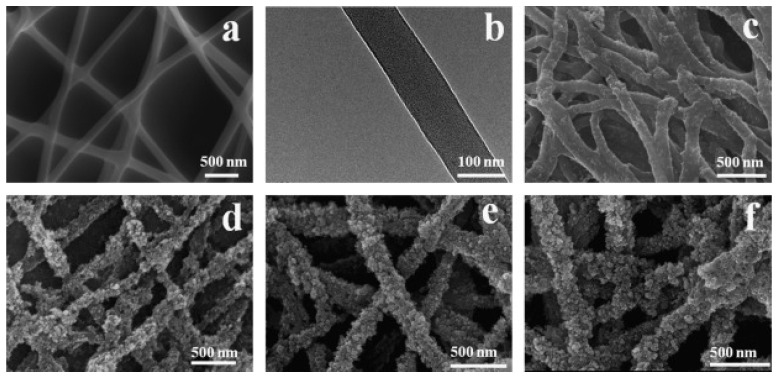
(**a**) SEM image of electrospun nanofibers of natural cotton cellulose, (**b**) TEM image of nature cotton cellulose nanofibers, and (**c**–**f**) SEM images of CC/CdS-5, CC/CdS-10, and CC/CdS-20, CC/CdS-30. (Reprinted from the work of Liu et al. [41]. Copyright 2015, with permission from Elsevier).

The use of natural cotton cellulose (CC) to prepare nanofibers decorated with CdS NPs and the study of their photocatalytic activity for the photodegradation of rhodamine B (RhB) was reported by Qiuyan Liu et al. [41]. The methodology to obtain the hybrid NFs combines the electrospinning technique and the chemical bath deposition. To prepare the cotton cellulose nanofibers, 1.15 wt.% cotton was added to 8.5 wt.% LiCl/DMAc solution under vigorously stirring at 80 °C for 4 h. The parameters used in the electrospinning process were: applied voltage of 18 kV, the syringe to collector distance 15 cm, humidity under 40%, flow rate of 0.8 mL/h, and room temperature. The CdS nanoparticles were formed in situ at the surface of cotton cellulose nanofibers. For this, CC nanofibers were immersed in 0.1 M Cd(NO_3_)_2_ ethanol solution for 3 min and were rinsed with deionized water twice. Then, the Cd^2+^ ion-loaded nanofibers were immersed into 0.05 M Na_2_S solution for 3 min, followed by a second rinsing. The cycle was repeated by 5, 10, 20, and 30 times. In between cycles, the sheet was kept drying at 60 °C for 5 min. Correspondingly, the obtained CC/CdS nanocomposites were labeled as CC/CdS-5, CC/CdS-10, CC/CdS-20 and CC/CdS-30. Figure 11 shows the SEM images of bare CC NFs and CC/CdS NFs.

In general, more agglomerated and larger CdS nanoparticles were deposited on the uniform and smooth surfaces of CC nanofibers as the number of cycles of CdS NPs synthesis increased. The obtained CC/CdS nanocomposites showed excellent photocatalytic efficiency to RhB dye degradation. After 40 min of visible light irradiation, the CC/CdS-20, CC/CdS-10, CC/CdS-30, CC/CdS-5, and CdS nanoparticles were able to degrade ca. 99%, 93%, 83%, 78% and 51% of the RhB dye, respectively. Additionally, the NFs decorated with CdS NPs exhibited remarkable photostability. Importantly, a slight loss of photocatalytic activity after three cycles of use was observed.

### 3.3. Electrospun Nanofibers of Copolymers and Polymer Blends Decorated with Photocatalytic Nanoparticles

Despite the use of copolymers and polymer blends to produce electrospun hybrid nanofibers could present several advantages, such as facilitating the electrospinning process and the combination of polymers or polymer building blocks with different structure and functional groups, which could confer different properties to the NFs; the studies reported to date are scarce. 

The preparation of hybrid nanofibers of poly (vinylidene difluoride-co-trifluoroethylene) (P(VDF-TrFE)) copolymer, containing TiO_2_ nanoparticles (P(VDF-TrFE)/TiO_2_) and TiO_2_/Graphene oxide nanocomposite (P(VDF-TrFE)/TiO_2_/GO), was reported by Almeida and coworkers [42]. To obtain these photocatalytic systems, in both cases (TiO_2_ and TiO_2_/GO NPs), different amounts of NPs were sonicated for 2 h with a P(VDF-TrFE) solution in N,N-dimethyl formamide/methyl ethyl ketone (85/15, *v*/*v*) to achieve a good dispersion. Later, the solution was kept under vigorously stirring for 2 h. Finally, the electrospinning process was carried out at 1.5 kV, flux of 0.5 mL/h, and the electrospun fibers were collected at 15 cm from the needle tip. The electrospun mats showed smooth fibers with the presence of beads. The authors attributed this to the low viscosity of the solution and a low concentration that does not allow the adequate entanglement of the polymer chains. Furthermore, the addition of TiO_2_/GO NPs would increase the solution electrical conductivity and, consequently, smaller fiber diameter due to the extra mechanical stretch promoted by the applied electrical field. This could explain that the number of beads present in the NFs mat increases with NPs addition, giving rise to a necklace-like fiber structure. SEM studies proved that bare fibers have an average fiber diameter of 518 ± 119 nm, while nanocomposite mats showed a decrease in the average fiber diameter varying from 318 ± 126 down to 226 ± 93 nm, for the samples from 3 to 8 wt.% of TiO_2_/GO, respectively.

The results obtained showed that GO incorporation substantially improves MB adsorption, reaction rate, and removal efficiency under UV and Visible radiation. Additionally, all the samples containing GO could remove 100% of MB in less than 90 min, under visible light irradiation. The authors attribute this improved photocatalytic activity to the high surface area and porosity of the electrospun samples and the advantageous electrical and structural properties of graphene oxide. It is likely that bead-shaped fiber mats could also enhance the photocatalytic activity for the degradation of organic pollutants under visible light irradiation due to the increase in light scattering and photoactive surface area.

**Figure 12:**
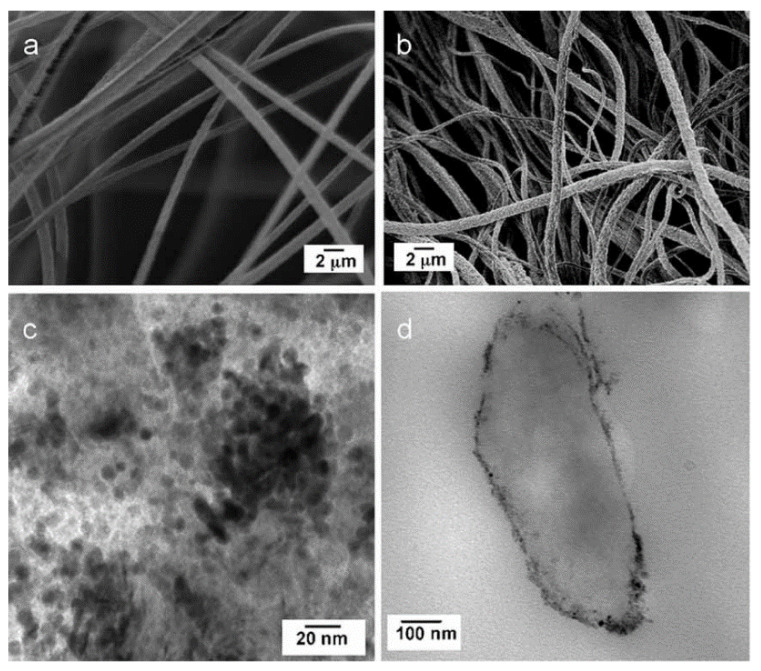
SEM images of (**a**) electrospun PSEI nanofibers and (**b**) TiO_2_ LBL-NF. TEM image of (**c**) TiO_2_ NPs and (**d**) the cross-section of TiO_2_ LBL-NF. (Reprinted from the work of Lee et al. [43]. Copyright 2010, with permission from John Wiley and Sons).

Random block copolymers, such as poly(dimethylsiloxane-block-etherimide) (PSEI), have also been used. Lee and coworkers [43] prepared nanofibers of this random block copolymer containing 35–40 wt.% dimethyl siloxane copolymerized with polyetherimide (PEI) units and modified their surface with TiO_2_ NPs by the layer by layer (LBL) technique. The copolymer nanofibers were obtained by the electrospinning of a 22 wt.% PSEI solution in a mixture of DMF and pyridine, the electrospinning parameters, voltage, solution flow rate, and the collector distance were set up to 30 kV, 0.01 mL min^−1^, and 35 cm, respectively. The authors indicate that the siloxane units provide NFs flexibility and resistance against ultraviolet-light-aging effect and variations in temperature. The average diameter of the PSEI fibers prior to coating, determined from SEM images (Figure 12a), was 650 ± 180 nm and the surface area determined by BET analysis was 12 m^2^ g^−1^. The LBL deposition of TiO_2_ NPs on electrospun fibers was carried out after the plasma treatment of PSEI. NFs. An electrostatically bonded coating on fibers was prepared by alternating dipping in (octa(3-ammoniumpropyl) octa- silsesquioxane octachloride) POSS-8NH_3_^+^ 8Cl^−^ and TiO_2_ dispersion. Ten bilayers of negatively charged colloidal TiO_2_ NPs and positively charged polyhedral oligomeric silsesquioxanes were LBL-assembled on the plasma-treated PSEI NFs mesh (TiO_2_ LBL-NFs). The amount of TiO_2_ on the PSEI fibers was determined using inductively coupled plasma-atomic emission spectroscopy; 16.9 µg mg^−1^ or 1.4 mg m^−2^ of the nanofibers were obtained. Transmission electron micrographs showed that POSS/TiO_2_ layers were uniformly deposited on the PSEI fibers (Figure 12b,d). Finally, hybrid NFs were tested in the photocatalytic degradation of Bisphenol A BPA, an additive used in the plastics industry, acting as an endocrine disruptor in living organisms. The decomposition of BPA with TiO_2_ LBL-NFs reached 89.3%, resulting in an 18.4% higher degradation compared to colloidal TiO_2_ NPs. The multilayer-assembled TiO_2_ nanofibers TiO_2_ LBL-NFs, in addition to exhibiting a higher degree of BPA decomposition, maintained first-order kinetics for over 12 h, while the photocatalytic activities of colloidal TiO_2_ NPs were initially higher; however, this behavior exhibited an abrupt decrease due to nanoparticles agglomeration. The authors determined that BPA adsorption on TiO_2_ LBL-NF was significantly higher than TiO_2_ NPs. The above was attributed to the increase in the binding affinity due to the additional adsorption sites presented by cationic species of polyhedral oligomeric silsesquioxanes POSS.

**Figure 13:**
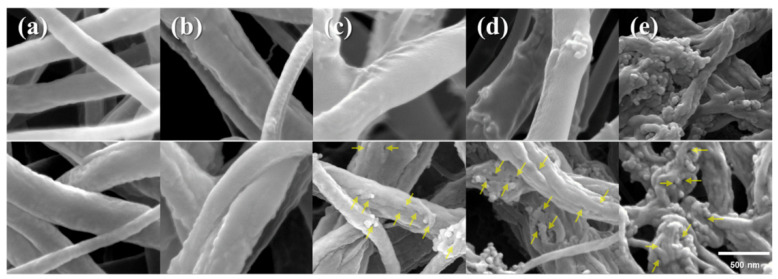
SEM image of the samples with different blending composition: (**a**) PVDF(18%), (**b**) PVDF(18%)-TiO_2_, (**c**) PVDF(12%)/PVP(6%)-TiO_2_, (**d**) PVDF(9%)/PVP(9%)-TiO_2_, and (**e**) PVDF(6%)/PVP(12%)-TiO_2_ (Top: before washing; Bottom: after washing). Arrows point to surface pores. (Reprinted from the work of Lee et al. [30]. Copyright 2018, with permission from American Chemical Society).

Poly (vinylpyrrolidone) (PVP), a synthetic polymer with a unique combination of relevant physical and chemical properties, soluble in water and many organic solvents, has also been used to obtain hybrid nanofibers with photocatalytic NPs. For example, the preparation of PVDF/PVP blend NFs containing TiO_2_ NPs has been reported [30]. To obtain the photocatalytic NFs, PVDF and PVP were dissolved at various ratios (PVDF(wt.%):PVP(wt.%) = 18:0, 12:6, 9:9, and 6:12) in DMAc/acetone (1:1 v:v along with TiO_2_ (4 wt.%). The solutions were vigorously stirred at 60 °C for 1 h and cooled to room temperature before the electrospinning process. The hybrid nanomaterial PVP acts as a sacrificial polymer to increase the surface area and improve light access to TiO_2_. To wash out the PVP and generate electrospun porous fibers, the electrospun mat was immersed in water, sonicated, and placed at 60 °C for 24 h. The blend composition showed directly affects the surface morphology and hydrophobicity of the electrospun porous fiber mat. The SEM images of the NFs with TiO_2_ adding and subsequent washing of the sacrificial PVP show NFs of rougher surface (Figure 13). The authors determined that PVP removal by washing to generate porous structure significantly increased the BET surface area, e.g., from 30.6 to 117.2 m^2^/g for PVDF(12%)/PVP(6%)-TiO_2_. Interestingly, TEM analysis showed that TiO_2_ was present in the fiber’s interior throughout the cross-section. For MB removal, the authors exploited the “bait-hook and destroy” strategy, wherein dye adsorption (bait-hook) and photocatalytic dye degradation (destroy) are carried out simultaneously. The strategy is based on using an appropriate support material that offers an active surface to adsorb and bring priority pollutants near the photocatalytic sites. Thus, short-lived ROS would be more efficiently exploited. As a result, the excellent adsorption of MB on the porous fiber mats was obtained, and then, the almost complete dye degradation was achieved using UV light irradiation for 120 min. Additionally, over 10 reusing cycles, there was no change in MB adsorption capacity and the photocatalytic degradation, reflecting high stability and reusability. The authors concluded that effective immobilization of TiO_2_ on polymers with an affinity for specific priority pollutants helps to increase the efficiency and reduce the energy requirements of photocatalytic water treatment. For example, the TiO_2_-embedded PVDF fiber mats’ hydrophobic surface would facilitate the adsorption and concentration of nonpolar organic contaminants near photocatalytic sites.

**Figure 14:**
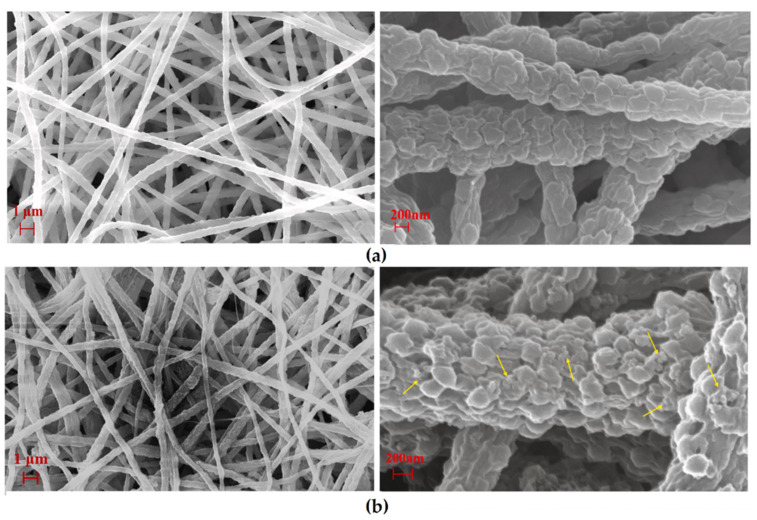
SEM of (**a**) PVDF electrospun nanofibers and (**b**) PVDF-TCN-2g electrospun nanofibers (arrows point to catalysis). With different magnification. (Reprinted from the work of Zheng et al. [44]. Copyright 2021, with permission from Elsevier).

Zheng and coworkers reported the same blend of PVDF/PVP to prepare a novel composite material with high photocatalytic activity starting with the nanofibers of the blend with the immobilized nanoparticles of the core–shell structure of the photocatalyst TiO_2_@g-C_3_N_4_ (TCN) [44]. For the preparation of the nanofibers, PVDF and PVP were dissolved in a DMAC/acetone solvent mixture (v:v = 1:1) in the ratio PVDF (wt.%):PVP (wt.%) = 8%:2%. The core–shell nanoparticles of TiO_2_ and the metal-free polymer n-type semiconductor graphite-like carbon nitride (g-C_3_N_4_) were added. The resulting solution was stirred at 60 °C for 2 h and subjected to ultrasound for 4 h to achieve good dispersion. The electrospinning conditions were: an applied voltage of 20 kV, a flow rate of 2 mL h^−1^, 15 cm between the needle and the collector, and a 1.07 mm blunt needle (G17) was used. Next, the electrospun nanofibers were immersed in deionized water and subjected to sonication to remove PVP. Finally, the NFs were dried under vacuum at 90 °C for 24 h. Figure 14 shows SEM images of PVDF and PVDF-TCN-2 g nanofibers. The PVDF nanofibers obtained showed uniform distribution and sizes, with diameters between 300–400 nm. Removing the sacrificial PVP produced a rougher surface with pore formation, increasing the specific area and facilitating interaction with the catalyst nanoparticles. For fibers prepared with 0.2 g of catalyst PVDF-TCN-2 g, SEM images show that the catalyst has a small effect on the nanofiber structure, with the nanofibers showing a slight increase in surface roughness. According to SEM elemental mapping scanning spectra for the fiber surface of carbon, fluorine, oxygen, and titanium, catalyst nanoparticles were located on the surface of nanofibers and in the pores. Atomic force microscopy measurements corroborated the higher roughness of the catalyst-containing nanofibers. Additionally, the authors established by water contact angle measurements on the nanofiber mats that the inclusion of the catalyst nanoparticles conferred high hydrophilicity to the surface.

The degradation of tetracycline, an oral antibiotic, was evaluated using PVDF and PVDF-TCN electrospun nanofibers under visible light irradiation. PVDF nanofibers showed no photocatalytic activity to degrade tetracycline. At the same time, for PVDF-TCN fibers, the degradation capacity increased up to 0.2 g catalyst content, reaching a degradation efficiency of 81.9%, 92.3%, and 97.0% after 300 min of irradiation for PVDF-TCN 0.1 g, PVDF-TCN 0.15 g, PVDF-TCN 0.2 g, respectively. For a TCN content of 0.3 g, the catalytic activity decreased. The adsorption of tetracycline on these substrates was also evaluated under dark conditions for 30 min. The adsorption capacity of PVDF fibers, PVDF-TCN 0.1 g, PVDF-TCN 0.15 g, PVDF-TCN 0.2 g and pure TCN were 3.97%, 4.95%, 9.17%, 16.42% and 12.9%, respectively. The above would indicate that the adsorption of tetracycline by both the polymer and the catalyst contribute to its degradation, facilitating the encounter of the drug molecules with the catalytic sites.

### 3.4. Electrospun Nanofibers of Polymers/Carbon Nanostructures Blends Containing Photocatalytic Nps

**Figure 15:**
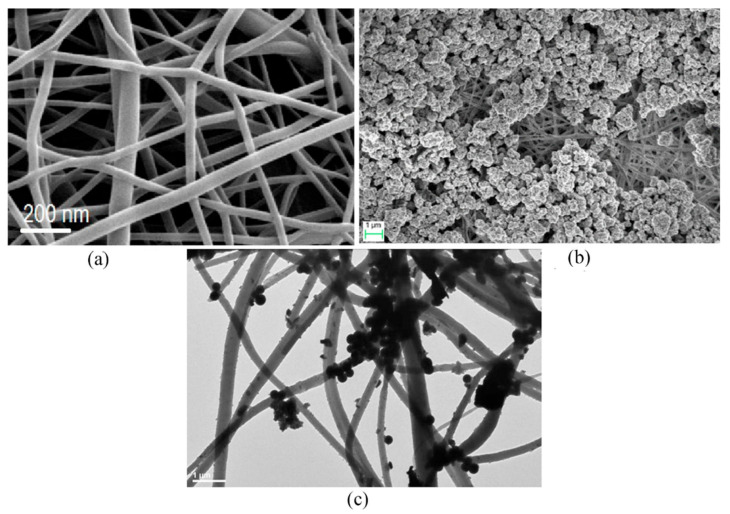
SEM images of (**a**) PAN-CNT (**b**) and PAN-CNT/TiO_2_-NH_2_, and TEM Images of (**c**) PAN-CNT/TiO_2_-NH_2_ nanofibers composite. (Reprinted from the work of Uheida et al. [29]. Copyright 2019, with permission from Elsevier).

According to the reviewed literature, another strategy that has proven to be effective in preparing hybrid nanofibers with photocatalytic properties for the degradation of water pollutants is incorporating carbon nanostructures in these materials. For example, Uheida and coworkers prepared polyacrylonitrile/multiwall carbon nanotubes composite nanofibers containing surface-modified TiO_2_ nanoparticles PAN-CNT/TiO_2_-NH_2_ [29]. Firstly, the solution to be electrospun was prepared by the addition of PAN/DMF solution to 3 wt.% of surface-activated CNTs in a DMF dispersion. The electrospinning process was carried out at room temperature with a voltage of 25 kV, the flow rate was set 0.5 mL/h, and the distance from the needle tip to the collector was 15 cm. The electrospun PAN/CNT mat was dried in a vacuum oven to remove the remaining amount of solvent and later was immersed into the crosslinking medium containing 2.5 wt.% glutaraldehyde (GA), kept shaking for 24 h at room temperature. After the activation reaction was completed, PAN/CNT composite nanofibers mat was removed and then immersed into 2 mL of an aqueous dispersion of TiO_2_-NH_2_ NPs and kept shaking for 24 h. Finally, the crosslinked composite nanofibers were washed with ethanol, deionized water, and dried in air at room temperature. The SEM and TEM images of the fabricated PAN-CNT/TiO_2_-NH_2_ composite nanofibers (Figure 15) show smooth nanofibers with an average diameter of 126 ± 4 nm and show that amino-functionalized TiO_2_ NPs are attached to the surface of the PAN/CNT nanofibers as a result of the crosslinking process. The PAN-CNT/TiO_2_-NH_2_ composite nanofibers were evaluated in the photocatalytic degradation of pharmaceuticals ibuprofen, cetirizine, and naproxen. Interestingly, the incorporation of CNTs enhanced the mechanical strength of the composite NFs and contributed as a photoactive phase (acting as an effective electron donor) in the photodegradation process of the studied drugs. Remarkably, the complete photodegradation of ibuprofen, naproxen, and cetirizine was achieved at 210, 90, and 50 min, respectively, under visible light irradiation. The photodegradation efficiency of PAN-CNT/TiO_2_-NH_2_ composite nanofibers remained stable during five sequential cycles, indicating good stability and reusability of the catalyst.

The same research group also reported the preparation of electrospun composites nanofibers of PAN and CNT and incorporating TiO_2_ NPs surface-modified TiO_2_-NH_2_ to generate PAN-CNT/TiO_2_-NH_2_ NFs. However, in this report, the authors studied and established the physicochemical parameters involved in phenol photodegradation in an aqueous solution [45]. The same methodologies were used for NFs preparation and modification. TiO_2_ NPs (diameters ranging from 5 to 30 nm) were strongly attached to the PAN-CNT NFs. At optimum conditions, a 99.8% phenol degradation in the presence of PAN-CNT/TiO_2_-NH_2_ and irradiation with a 100 W halogen lamp as the visible light source was achieved in a time lesser than 20 min.

Sharma and coworkers used graphene oxide (GO) to prepare GO/PAN composite nanofibers by electrospinning. The GO/PAN NFs were later modified with TiO_2_ NPs, and finally, their performance in the photodegradation of Rhodamine 6G (Rh6G) dye was studied [46]. The solution for electrospinning was prepared by stirring a mixture of PAN (1.75 g), GO (20 mg), and DMF (20 mL) for 24 h. A syringe with a 0.5 mm inner diameter capillary, a high potential of 13 kV, a distance of 15 cm between the syringe and the collector, and a flow rate of 0.3 mL/h were used for electrospinning. The incorporation of TiO_2_ nanoparticles into PAN/GO nanofibers was achieved by immersion in a TiO_2_ sol–gel solution for 15 min. Finally, the nanofibers were dried at 65 °C for 1 h in air. FTIR and UV-Vis spectroscopy confirmed the strong interaction between the nanofibers and rhodamine. PAN/GO composite nanofibers showed a good photocatalytic effect on rhodamine degradation; 65% of dye was degraded after 12 h under natural sunlight with only 6 mg addition of PAN/GO nanofibers in 20 mL of dye solution, while with TiO_2_-loaded PAN/GO nanofibers, the photocatalytic degradation of the dye was enhanced with UV irradiation compared to visible light. Additionally, the TiO_2_ loading on the nanofiber shows a faster degradation of the dye with UV light compared to visible light.

**Figure 16:**
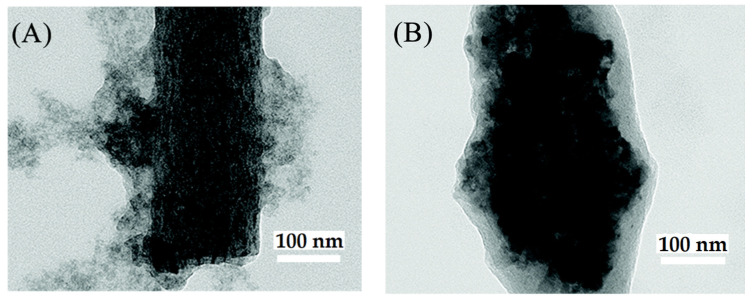
TEM images of (**A**) TiO_2_@GO and (**B**) TiO_2_@GO@C nanofiber membranes. (Adapted from the work of Zhang et al. [47]. Copyright 2020, with permission from Royal Society of Chemistry).

On the other hand, an interesting way to obtain carbon nanofibers containing photocatalytic nanoparticles starting from polymer nanofibers has been used. In this case, the polymer NFs, after being obtained, were subjected to thermal treatments to produce the calcination of the polymer and carbon generation. A fascinating example is the work of Zhang and coworkers, in which they report the obtaining of peapod-like electrospun nanofiber membranes, consisting of TiO_2_ NPs, graphene oxide and carbon, (TiO_2_@GO@C NFs), which showed excellent efficiency in the photocatalytic degradation of methylene blue [47]. To fabricate the TiO_2_@GO@C electrospun nanofiber membranes, a solution of the TiO_2_ precursor titanium isopropylate TTIP, graphene oxide and PAN was prepared in DMF using magnetic stirring and ultrasound for homogenization. The solution was then electrospun using a syringe with a 0.6 mm diameter needle, 13–15 kV and 13.5 cm tip-collector distance. After 12 h of ripening, the membranes were preoxidized at 280 °C for 4 h. Finally, the membranes were carbonized at 600–900 °C under an Ar atmosphere with a flow rate of 200 mL min^−1^, using a heating rate of 5 °C min^−1^. Using this process, TiO_2_-precursor@GO@PAN nanofiber membranes form TiO_2_@GO@C peapod-like nanofiber membranes. Figure 16 shows TEM images of (A) TiO_2_@GO and (B)TiO_2_@GO@C nanofiber membranes. It is observed that the TiO_2_ NPs are almost wrapped by GO forming a pea-shaped TiO_2_@GO structure. Moreover, in Figure 16B, it can be observed that TiO_2_@GO is wrapped with the carbon nanofiber, forming peapod-shaped TiO_2_@GO@C nanofiber membranes. The excellent photocatalytic degradation efficiency of MB, 98.5% over 3 h was obtained using peapod-like TiO_2_@GO@C nanofiber membranes with 0.3 wt.% GO. The authors ascribed these promising results to the large surface area, along with abundantly generated functional groups and excellent thermal conductivity of GO that improve the transport of carriers generated by TiO_2_ NPs, in photocatalysis. Additionally, good mechanical properties are provided by the inclusion of a pea-like TiO_2_@GO structure, which shows a strengthening effect in the carbon nanofibers, improving their resistance to fracture.

**Figure 17:**
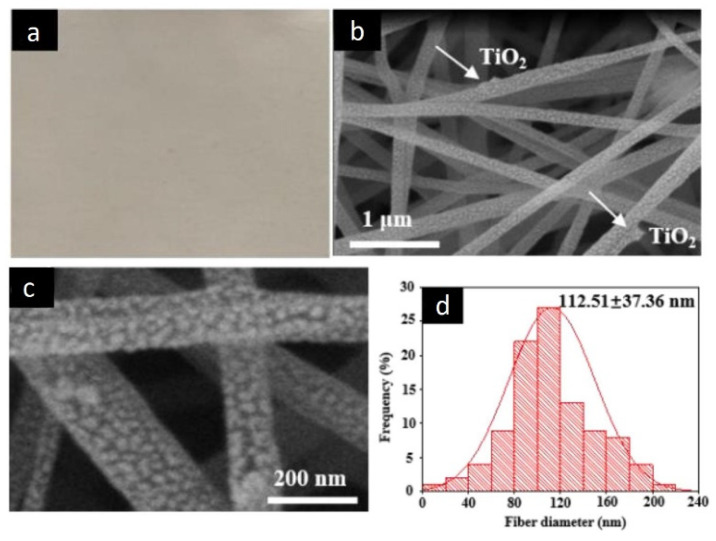
Optical imaging (**a**), typical SEM (**b**), FESEM image (**c**), and histogram (**d**), of PAN/B-cyclodextrin/TiO_2_/GOv with TiO_2_:GO 9:1 nanofiber diameter distribution. (Adapted from the work of Zhang et al. [48]. Copyright 2021, with permission from Elsevier).

Another interesting report describes obtaining process of a porous nanofibrous membrane consisting of PAN, β-cyclodextrin, TiO_2_ NPs and graphene oxide, PAN/b-CD/TiO_2_/GO, and the evaluation of this material in the photocatalytic degradation of methyl orange and methylene blue under natural sunlight [48]. In this work, the solution for electrospinning was prepared by mixing TiO_2_ NPs and graphene oxide in different proportions in DMF; then, the mixture was subjected to an ultrasonic bath; subsequently, PAN and β-cyclodextrin were added, and the mixture was stirred up to complete dissolution. To obtain electrospun NFs, a syringe with a 0.7 mm needle, voltage set at 18 kV, flow rate at 0.4 mL/h and 18–20 cm between the needle and the manifold were used. Apparently, PAN nanofibrous membranes trended to plasticize and swell in aqueous environments, limiting their ability to adsorb contaminants. For this reason, PAN is often combined with other materials for applications in aqueous media in order to moderate hydrophilic/hydrophobic balance. Specifically, the incorporation of β-cyclodextrin (β-CD) would include a hydrophobic cavity and a hydrophilic outer wall. Figure 17 shows that the nanofiber membrane has darkened areas due to the presence of GO. SEM images show rough nanofibers due to the presence of TiO_2_ NPs and GO clusters. As the amount of GO incorporated increases, there is a decrease in the average diameter of the NFs, an effect that was attributed to the increase in the conductivity of the electrospun solution. TiO_2_NPs and GO were uniformly dispersed in the PAN/β-CD nanofibrous membranes without showing aggregation. The PAN/β-CD/TiO_2_/GO nanofibers showed good stability and reusability in MB and MO adsorption and photodegradation tests. During the first three cycles of use, the degradation efficiency of MB and MO by the nanofibrous membranes remained at 80%, then decreased by approximately 10% in the fifth cycle. The authors attribute the good efficiency obtained to the low density of PAN and the porosity of the PAN/β-CD/TiO_2_/GO nanofibrous membranes, which allow their flotation in the dye solution, increasing the contact surface and facilitating light penetration.

## 4. Concluding Remarks

A wide range of polymers have been proposed to obtain hybrid materials of polymeric nanofibers/photocatalytic nanoparticles with potential applications in the light-driven removal of organic pollutants from water. Most of the studies carried out to date exploited synthetic polymers, and only in a few cases natural polymers, biopolymers, or biodegradable polymers were considered. This is probably due to the difficulty of finding an adequate compromise between polymer degradation and the durability of the photocatalytic material during use. In our opinion, future designs should consider the increased use of naturally occurring polymers. In this perspective, blending the materials with other environmentally friendly polymers (such as PCL, PLA PVP, and PEO, among others) may be helpful for the processing of the natural polymers. The blending strategy can be also exploited to modify and improve the properties of natural polymers (such as mechanical properties, conductivity, hydrophobicity, etc.). Among the different polymers that have been used in hybrid photocatalytic materials, we believe that optically active polymers (conductive, donor-acceptor, -conjugated) could also be considered as interesting options since these types of polymers are expected to contribute actively to the photodegradation of pollutants and/or enhance the effect of the light.

A relevant aspect that should be considered, in our view, is the role played by the polymeric electrospun nanofibers, which extends much further than simply being a support for the photocatalytic material. In fact, the polymer can actively contribute to enhancing the adsorption of pollutants, facilitating their approach to the photocatalytic sites or it can interact with the surface of the catalytic hybrid material to improve its performance. An outstanding example of the possible “active” role of polymers is represented by cellulose acetate NFs, which, thanks to the presence of carboxylic acid groups, bind very efficiently to photocatalytic titania nanoparticles. Hence, using this polymeric precursor, it was possible to obtain a photocatalytic hybrid material based on polymeric nanofibers with excellent performance and durability. Another relevant strategy for designing photocatalytic hybrid materials exploits a matrix composed of nanofibers of a polymer blend. In this case, high porosity in the nanofibers can be achieved by removing one of the polymers, for example, with a specific solvent. As an advantage, the enhanced porosity significantly increases the contact area and the availability of photocatalytic sites, leading to a considerable improvement of the photocatalytic performance of the material. An alternative approach to optimize photocatalytic activity is the calcination of hybrid polymer nanofibers to obtain carbon nanofibers or to produce a one-dimensional structure of semiconductor crystals. The interaction of the catalytic nanoparticles with the included carbon nanostructures, such as graphene derivatives and carbon nanotubes, yields highly performing materials.

Regarding the final application of the hybrid electrospun systems reviewed in this review, we would like to point out that important challenges still remain, and more issues should therefore be fixed, such as the control of the fouling and the increase in the permeate fluxes that are still relatively low. In addition, more investigations are needed to demonstrate the durability of the membranes in terms of photocatalytic activity, as well as the cost-effective synthesis of these hybrid nanomaterials for large scale applications.

## Figures and Tables

**Table 1:** Electrospun polymers, solvents used and carrier if corresponding. Adapted with permission from Xue et al. [23]. Copyright © 2019, American Chemical Society.

Material	Solvent	Carrier Polymer
**Thermoplastic polymer**		
Nylon-6	Formic acid and acetic acid	
PAN	DMSO and trifluoroacetic acid	
PCL	HFIP	
PEO	Water	
PLA	DMF and chloroform	
PLGA	THF and DMF	
PP		
PS	DMF and toluene	
PVC	THF and DMF	
PVP	Ethanol and water	
**Thermoset polymer**		
Bisphenol A ethoxylate dimethacrylate		PCL
Epoxy resin	Ethanol and acetone	PVP
Ethylene/propylene/diene terpolymer rubber		
PDMS	THF	PVP
Conjugated polymer		
PANi	Formic acid	
PPy	DMF	
PEDOT and PSS	Water	
**Natural polymer**		
Chitosan	Trifluoroacetic acid	
Collagen	HFIP	
Gelatin	Trifluoroethanol and HFIP	
Hyaluronic acid	DMF and water	
Silk fibroin	Formic acid	
**Metal**		
Ag	Ethylene glycol	
Ag	Water	PVA
Co	Isopropyl alcohol and water	Polyvinyl butyral
Cu	Water	PVA
Fe	Isopropyl alcohol and water	poly(vinyl butyral)
Pt	DMF and water	PVP
Pt–Au	DMF and water	PVP
**Metal oxides**		
Al_2_O_3_	Ethanol	PVP
CeO_2_	Water	PVA
Co_3_O_4_	DMF	PVP
CuO	Water	PVA
Fe_2_O_3_	Water	PVA
Mn_3_O_4_	DMF and chloroform	PMMA
SiO_2_	Water and ethanol	
SnO_2_	Water, propanol, and isopropanol	PVA
TiO_2_	Ethanol	PVP
V_2_O_5_	DMF and chloroform	PMMA
WO_3_	Propanol and DMF	poly(vinyl acetate)
ZrO_2_	Ethanol	PVP
BaTiO_3_	Isopropanol	PVP
CoFe_2_O_4_	DMF and THF	poly(vinyl acetate)
LiCoO_2_	Water	
NiFe_2_O_4_	Isopropanol	PVP
ZnCo_2_O_4_	Ethanol	PVP
LiNi_0.5_Mn_1.5_O_4_	Ethanol	PVP
**Metal nitrides**		
Li_3_N	Water	PVA
NbN	Ethanol	PVP
TiN	Ethanol and acetic acid	PVP
VN	DMF	PVP
Ti–V–N	2-propanol	PVP
**Metal carbides**		
Mo_2_C	Water	PVA
SiC	DMF	PS
TiC	DMF	PVP
WC	Water	PVP
ZrC	Ethanol	PVP
**Doped carbon**		
Ni/N,S-doped carbon	DMF	PAN
CoSe/N-doped carbon	DMF	PAN
Fe_3_O_4_/N-doped carbon	DMF	PAN
SnS_2_/N,S-doped carbon	DMF	PVP
WS_2_/N-doped carbon	DMF	PAN
MnCo_2_O_4_@N-doped carbon	DMF	PAN

## Data Availability

Not applicable.
